# Non-classical immune checkpoint CD137/CD137L and CD200/CD200R expressions are regulated by the tumor immune microenvironment in lymph node aspirates from lung cancer patients

**DOI:** 10.3389/fimmu.2026.1766726

**Published:** 2026-05-26

**Authors:** Iwona Kwiecień, Agata Raniszewska-Borys, Elżbieta Rutkowska, Rafał Sokołowski, Karina Jahnz-Różyk, Piotr Rzepecki

**Affiliations:** 1 Department of Internal Medicine and Hematology, Laboratory of Hematology and Flow Cytometry, Military Institute of Medicine-National Research Institute, Warsaw, Poland; 2 Department of Internal Medicine, Pneumonology, Allergology, Clinical Immunology and Rare Diseases, Military Institute of Medicine-National Research Institute, Warsaw, Poland; 3 Department of Internal Medicine and Hematology, Military Institute of Medicine-National Research Institute, Warsaw, Poland

**Keywords:** CD137/CD137L, CD200/CD200R, EBUS/TBNA, flow cytometry, immune checkpoints, lung cancer, lymph nodes aspirates, PD-L1/PD-L2

## Abstract

**Background:**

Immune escape is a hallmark of lung cancer, and limited responsiveness to PD-1/PD-L1 blockade highlights the need to identify additional immunoregulatory mechanisms in the mediastinal tumor microenvironment (TME). Non-classical immune checkpoints, including CD137/CD137L and CD200/CD200R, may interact with classical pathways but remain insufficiently defined in metastatic lymph nodes (LNs). We evaluated their expression on tumor cells and lymphocytes and their associations with PD-L1 and PD-L2.

**Methods:**

LN aspirates obtained during transbronchial needle aspiration biopsy (EBUS/TBNA) from 71 lung cancer patients were analyzed using cytology, hematological screening, and flow cytometry. Expression of CD137, CD137L, CD200, CD200R, PD-1, PD-L1, PD-L2 was assessed on tumor cells and lymphocytes.

**Results:**

Tumor cells showed high EpCAM and cytokeratin expression. CD137L was mainly detected on tumor cells, whereas CD137 was predominantly expressed on CD8+ T lymphocytes. CD200 expression was higher on tumor cells, while CD200R was enriched on lymphocytes, particularly CD8^+^ T cells. Correlation analysis revealed significant associations between non-classical and classical checkpoints, including positive correlations of CD200R and CD137 on tumor cells with PD-L2, and a positive correlation between CD137 on lymphocytes and PD-1.Checkpoint expression varied with histology:: NSCLC exhibited higher PD-L1, PD-L2, and CD137 expression. Tumor-rich LNs showed reduced tumor-cell expression of these markers, while lymphocyte profiles remained relatively stable.

**Conclusion:**

CD137/CD137L and CD200/CD200R interact with PD-1–related pathways in metastatic LNs, forming an integrated immunoregulatory network predominantly shaped at the tumor cell level. Flow cytometric analysis of LN aspirates may help identify additional therapeutic targets in lung cancer.

## 1 Introduction

Lung cancer remains the leading cause of cancer-related mortality worldwide, accounting for approximately 1.8 million deaths annually, with non-small cell lung cancer (NSCLC) representing nearly 85% of cases (1). Despite advances in molecular profiling and targeted therapies, immune checkpoint inhibitors (ICIs) targeting the PD-1/PD-L1 axis remain a cornerstone of modern lung cancer treatment. Although these therapies have improved outcomes for select patient groups, primary resistance occurs in up to 70–80% of patients, while many initial responders develop acquired resistance (2, 3). Resistance to ICIs is now recognized as a multifactorial process involving both tumor-intrinsic and microenvironmental mechanisms. The mechanisms underlying this resistance extend far beyond PD-1/PD-L1 expression and include antigen presentation defects, T-cell exhaustion, altered tumor metabolism, and the presence of immunosuppressive stromal and myeloid cells (4).

Given the clinical limitations of PD-1/PD-L1 blockade, attention has increasingly turned to non-classical immune checkpoints, such as CD137/CD137L (4-1BB/4-1BBL) and CD200/CD200R, which contribute to the regulation of T-cell activation, NK-cell cytotoxicity, and myeloid cell function (5, 6). CD137 is a potent co-stimulatory receptor belonging to the TNFR family, typically expressed on activated T cells, NK cells and dendritic cells (7). Its ligand, CD137L, is expressed on antigen-presenting cells but has also been found on some tumor cells, where it may paradoxically contribute to immune dysregulation rather than activation (8, 9). Agonistic targeting of CD137 has shown promise in enhancing anti-tumor immunity, but CD137 signaling may be highly context-dependent within the tumor microenvironment (TME) (5). In leukemia and lymphoma, CD137 expression can promote malignant cell growth and survival and inhibit T cell activation (10, 11), suggesting that the overall effect of CD137 expression in tumor cells may be immunosuppressive. However, it is unclear whether such an immunosuppressive function can also be observed in solid tumors. On the one hand, expression of CD137 on the tumor cell surface could potentially compete with CD137 on T cells in binding CD137L on the surface of APCs, thereby suppressing the immune response against cancer. On the other hand, interaction of CD137 on the surface of tumor cells with CD137L on APCs could potentially activate or promote the immune response (6).

In contrast, the CD200/CD200R axis mediates immunoinhibiting through interaction with CD200R on myeloid cells, promoting a tolerogenic phenotype and suppressing anti-tumor immunity (12). Overexpression of CD200 or CD200R has been reported in multiple malignancies, including leukemia, melanoma, and lung cancer, and is associated with poor prognosis and accelerated disease progression (13–17). Overexpression of CD200 family members has been linked to poor clinical outcomes in multiple hematologic and solid tumors, including lung cancer (18). Notably, however, experimental data indicate that in certain contexts CD200 expression may exert anti-tumor effects, as demonstrated in a murine melanoma model where CD200 limited tumor growth and metastasis (19). Recent evidence in NSCLC suggests that high tumoral CD200 expression may even be associated with better survival, whereas CD200R correlates with poorer outcomes, indicating a potentially bidirectional and context-dependent role for the CD200/CD200R axis in lung cancer (18). At the same time, CD200-mediated regulation may contribute to the development of an immunosuppressive niche in lymph nodes (LNs), yet its overall function within the lung cancer TME remains incompletely defined.

Mediastinal LNs are central hubs of tumor–immune interaction and represent one of the earliest sites of immune modulation and metastatic spread in lung cancer. The TME within these nodes is highly dynamic, shaped by interactions between tumor cells, infiltrating lymphocytes, and antigen-presenting cells. Access to these anatomical regions via traditional biopsy methods is limited; however, endobronchial ultrasound–guided transbronchial needle aspiration (EBUS/TBNA) provides a minimally invasive, real-time method to obtain LN aspirates for diagnostic and immunologic assessment (20, 21). Flow cytometry of EBUS-derived LN aspirates has been shown to reliably characterize, among other things, leukocyte subpopulations, including T and B lymphocytes and expression of costimulatory molecules such as PD-1, PD-L1, and CTLA-4 (22–26). Our recent studies confirm that EBUS-TBNA samples offer robust utility for in-depth immune profiling; however, this method has rarely been used to study non-classical novel immune checkpoint pathways, which remain insufficiently understood in this anatomical niche.

Given the potential role of CD137/CD137L and CD200/CD200R in shaping the immune landscape and influencing therapeutic outcomes, we sought to characterize their expression on tumor cells and lymphocytes within EBUS/TBNA-derived LN aspirates from lung cancer patients. We further examined their relationship with PD-L1 and PD-L2 to determine whether these pathways operate independently or as components of an integrated immunosuppressive network.

The present study aimed to evaluate the expression of CD137/CD137L and CD200/CD200R on tumor cells and lymphocytes in EBUS/TBNA aspirates from lung cancer patients and to investigate their relationship with classical immune checkpoints PD-1, PD-L1 and PD-L2. Patients were then stratified by histological subtype and tumor cell fraction in LN aspirations to determine whether histologic type and tumor burden modulated the expression of the checkpoint pathways studied.

Understanding whether these pathways interact in the mediastinal TME may help identify new therapeutic targets for patients.

## 2 Material and methods

### 2.1 Patients

The study population consisted of patients referred for diagnosis of suspected lung malignancy. Individuals who had previously received anticancer treatment, showed clinical evidence of infection, had known autoimmune disorders, or were undergoing immunosuppressive therapy were excluded. After applying these criteria, 71 patients with histologically verified primary lung cancer and metastatic involvement of mediastinal LNs were ultimately enrolled. Tumour classification followed current WHO histopathological standards (27). All participants provided written informed consent for diagnostic procedures, including EBUS/TBNA aspiration. The study protocol was approved by the Ethics Committee of the Medical Chamber in Warsaw (KB/1441/23). Chest computed tomography was performed in all cases before EBUS/TBNA to guide LN selection.

During bronchoscopy, LNs with radiological or ultrasound features suggestive of metastasis were sampled systematically. Cytological material obtained via EBUS/TBNA was processed to generate cell blocks, which served as the basis for establishing the final diagnosis of nodal metastasis. Each aspirate was evaluated by an experienced pathologist to confirm diagnostic adequacy. The same aspirates were simultaneously allocated for multiparameter flow cytometric analysis in our laboratory, allowing parallel immunophenotyping of the LNs microenvironment.

LNs were classified as metastatic when cytopathological evaluation of the cell block confirmed the presence of malignant cells. All samples were collected at the time of the initial diagnostic (“baseline evaluation”) and were not reused for subsequent staging procedures. An overview of patient characteristics is provided in **Table 1**.

**Table 1:** Patients’ characteristic.

Characteristic	All patients	
Number of patients, n	71	
Age, years (mean ± SD)	67.6 ± 7.5	
Sex (female/male), n (%)	35 (49.3%)/36 (50.7%)	
Smoking status, n (%) - Current/former smoker - Never smoker	52 (73.2%) 19 (26.8%)	
Clinical stage I/II/III/IV, n	2/3/33/33	
Type of sample	EBUS–TBNA LN aspirates	
Distribution of LN stations, n	4R= 7, 4L = 7, 7 = 6, 10L = 1, 11R = 1, 11L = 2, mass= 7	
Tumor cells percentage in flow cytometry (% mean ± SD)	57.0 ± 35.0	
Tumor cells count in flow cytometry (k/µl mean ± SD)	53488 ± 95776	
Comparison: NSCLC vs SCLC (based on cell block)	NSCLC	SCLC
Number of patients	38	33
Age (mean ± SD years)	69.2 ± 8.8	67.3 ± 8.2
Sex f/m (n)	17/21	18/15
Histological subtypes:		
Squamous cell carcinoma SCC, n (%)	9 (23.7%)	–
Adenocarcinoma ADC, n (%)	22 (57.9%)	–
NOS, n (%)	6 (15.8%)	–
Large cells carcinoma, n (%)	1 (2.6%)	–
Comparison: Tumor cell fraction groups (by flow cytometry)	≤ 15% tumor cells	> 15% tumor cells
Number of patients	16	55
Age (mean ± SD years)	69.7 ± 5.7	67.0 ± 7.9
Sex f/m, n	8/8	27/28
Histological subtypes: NSCLC/SCLC, n	16/0	22/33

f, female; m, male; n; number; LN, lymph node; NSCLC, non-small cell carcinoma; SCLC, small cell carcinoma; SCC, squamous cell lung carcinoma; ADC, lung adenocarcinoma.

### 2.2 Materials

LN aspirates were obtained during routine EBUS/TBNA performed for the diagnostic evaluation of lung cancer. Material was collected from LNs at stations 4R, 4L, 7, 10L, 11R, and 11L, as well as from combined nodal–tumor masses when present. After completion of the diagnostic aspiration, an additional aliquot of approximately 1 mL was collected for laboratory analysis. Each aspirate was diluted in 0.9% sodium chloride and transferred to K_2_EDTA tubes; samples were subsequently assessed using a hematology analyzer and then processed for flow-cytometric evaluation at the Laboratory of Hematology and Flow Cytometry, Military Institute of Medicine–National Research Institute (WIM-PIB).

Cytological material from the same LNs was used to prepare cell blocks which were evaluated in the Department of Pathomorphology WIM-PIB, to confirm the presence or absence of malignant cells. Only patients with a cell block-based diagnosis of cancer in the analyzed LN were included in the final group. Cytopathological cell block evaluation was performed in parallel for all analyzed LN to confirm metastatic involvement; however, quantitative tumor-cell percentage assessment was based exclusively on multiparametric flow cytometry. For 31 lymph-node samples, quantitative cytopathological estimates of tumor-cell percentage were additionally available, primarily to assess their suitability for downstream molecular testing, and this subset was used for comparative evaluation of tumor-cell fractions. The LN aspirates obtained via EBUS/TBNA provided sufficient cellularity for further immunophenotyping, including assessment of tumor cells and local leukocyte populations.

All samples were collected between July 2023 and mid-2025 as part of routine diagnostic procedures at the Department of Internal Medicine, Pneumonology, Allergology, Clinical Immunology and Rare Diseases WIM-PIB, and then transmitted and analyzed in the hematological laboratory. The collected material consisted exclusively of aspirates obtained during the initial diagnostic, and no samples were reused for subsequent clinical evaluation. Only patients with cell block-confirmed malignancy in the analyzed LN were included.

### 2.3 Methods

#### 2.3.1 Hematological analysis

LN aspirates obtained during EBUS/TBNA were assessed using a Sysmex XN-1500 hematology analyzer (Sysmex Corp., Kobe, Japan) to verify whether the material was suitable for subsequent flow-cytometric evaluation. The analyzer allowed rapid quantification of total cellularity in each sample, which in our cohort consistently exceeded 2–500 cells/µl, indicating that the aspirates contained enough intact cells for reliable immunophenotyping.

**Figure 1:**
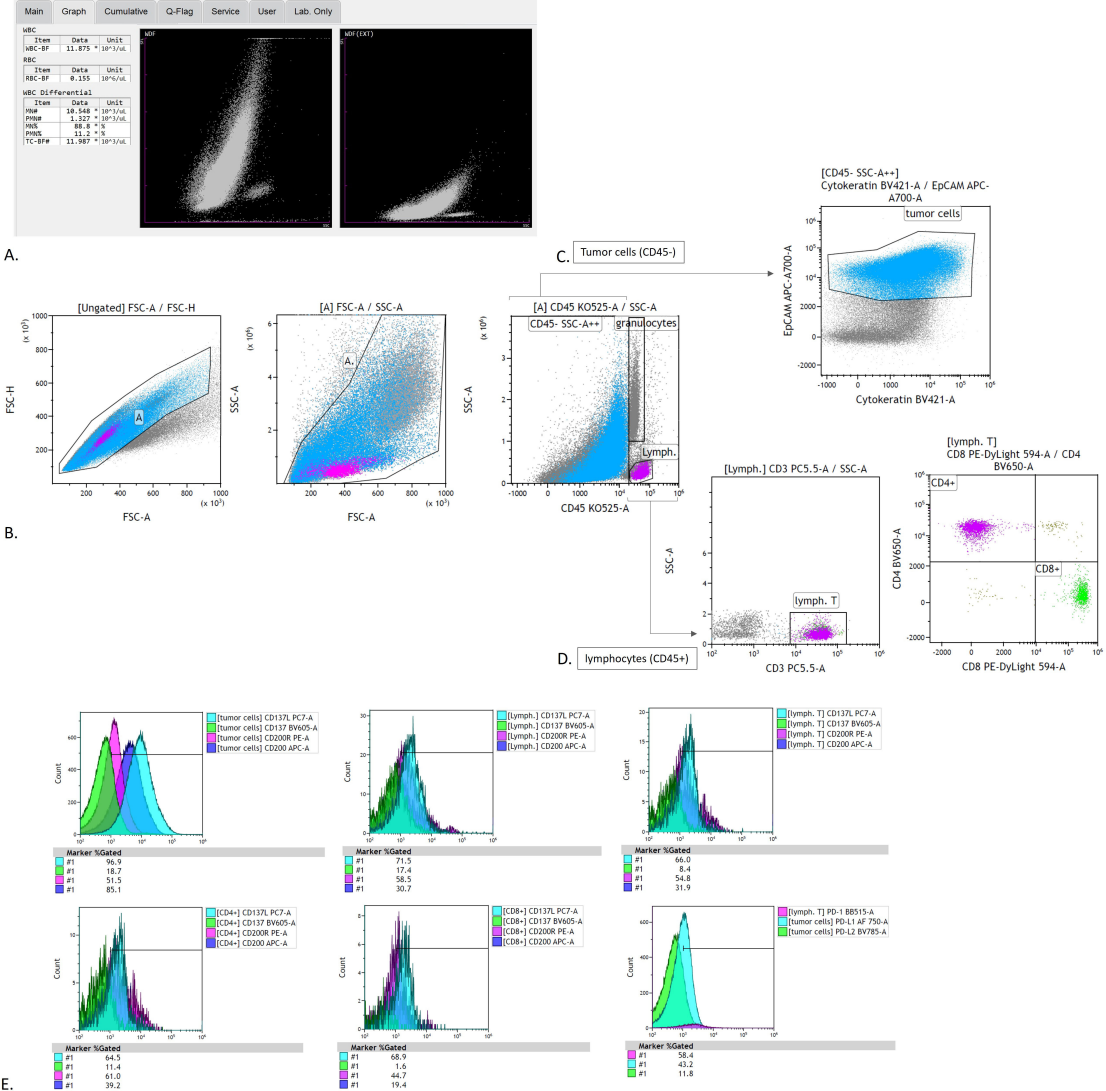
Representative figures from hematological analyzer and flow cytometry gating strategy of analyzing cells. **(A)** Scatter plot: SSC vs. SFL of the WDF and WDF (EXT) channels on the Sysmex XN analyzer, showing the distribution of highly fluorescent cells (high SFL, likely cancer cells) in lymph node aspirates. **(B)** Flow cytometry gating strategy. Doublets were excluded using FSC-A vs FSC-H, followed by removal of debris and non-cellular events using FSC-A vs SSC-A. Cells were then separated based on CD45 expression into CD45− tumor cells and CD45+ lymphocytes using a CD45 vs SSC-A plot. **(C)** Tumor cells were further identified within the CD45− population based on EpCAM and cytokeratin expression. **(D)** Lymphocytes (CD45+) were gated as CD3^+^ T cells and subsequently subdivided into CD4^+^ and CD8^+^ T cell subsets. **(E)** Representative histograms illustrating the distribution of selected immune checkpoint markers across tumor cells and lymphocyte subsets. Detailed gating strategy and threshold determination using fluorescence minus one (FMO) controls are provided in **Supplementary Figure 1**.

In addition to basic cell counts, the instrument provides fluorescence-based scatter parameters that reflect cellular size, granularity, and nucleic-acid content. These measurements enabled a preliminary assessment of the cellular composition of each aspiration. In many samples, a distinct cluster of highly fluorescent cells was visible in the SFL-SSC plot. This characteristic pattern (presented in **Figure 1A**), positioned high on the SFL axis, has previously been associated with cells exhibiting increased metabolic activity, and in our experience often corresponded to tumor-rich aspirates (24). Thus, although the hematology analyzer was not intended to confirm diagnosis, it enabled rapid analysis of samples likely to contain metastases and provided information on subsequent flow cytometry results.

#### 2.3.2 Flow cytometry methods

Multiparameter flow cytometry DxFLEX (Beckman Coulter Company, Marseille Cedex 9, France) assessed expression of:

o Immune cell markers: CD45, lymphocyte T gating: CD3, CD4, CD8
o Malignancy markers: EpCAM, cytokeratin
o Checkpoint molecules: CD137, CD137L, CD200, CD200R, PD-1, PD-L1, and PD-L2

Detailed information on antibodies used for flow cytometry, including marker, fluorochrome, clone, vendor, and analytical purpose and, is provided in **Supplementary Table 1**. Tumor cells were identified based on high EpCAM/cytokeratin expression, Light-scatter characteristics (high SSC-A expression) and CD45 negativity. Lymphocytes were gated as CD45^+^SSC-low cells with expression of CD3 (lymphocytes T) and with expression of CD4 (lymphocytes T CD3+ CD4+) or CD8 (lymphocytes T CD3+ CD8+). A minimum of ≥100,000 events per sample were acquired after exclusion of debris, doublets, and non-cellular events. The gating strategy used for the identification of these subpopulations is presented in **Figures 1B–D**. The median fluorescence intensity (GMF) and the percentage of positive checkpoint molecules on cells were assessed. The expression of checkpoint molecules (CD137, CD137L, CD200, CD200R, PD-1, PD-L1, and PD-L2) was assessed using single-parameter histograms generated for tumor cells and lymphocyte populations (**Figure 1E**). Positive expression was defined using fluorescence-minus-one (FMO) controls for each antibody, with gates set at the upper limit of the FMO signal and applied uniformly across all samples. Representative FMO-based gating examples for tumor cells and lymphocytes are shown in **Supplementary Figure 1**. Histograms are presented separately for each marker to illustrate FMO-derived gate placement and the general distribution of fluorescence intensity within each cell population. These plots serve to demonstrate the gating strategy rather than to provide quantitative comparisons between markers or fluorochrome channels. Cell viability in freshly processed EBUS/TBNA aspirates routinely exceeded 95% based on internal laboratory validation of diagnostic samples, supporting the suitability of the material for reliable immunophenotyping. Debris and non-cellular events were excluded using FSC-A vs SSC-A gating, followed by doublet discrimination (FSC-A vs FSC-H) (**Figure 1B**). Although viability staining was not included in the 13−color research panel due to channel limitations, EBUS−TBNA samples processed in our laboratory routinely undergo 7−AAD–based viability validation as part of our diagnostic workflow.

#### 2.3.3 Quality control and standardization of flow cytometry methods

Quality control procedures were implemented throughout the study. Daily cytometer performance was monitored using DxFLEX Daily QC Fluorospheres (ref. C39283). Fluorescence intensity standardization and long-term signal stability were ensured using Flow-Set Pro Fluorospheres (ref. A63492). The flow cytometry laboratory operates under routine clinical diagnostic standards and participates in external quality assessment programs, supporting the reliability and reproducibility of multiparametric immunophenotyping performed on EBUS/TBNA-derived samples. FMO controls were prepared for each marker and used consistently to define positive populations across all samples, minimizing inter-sample variability in gate placement.

#### 2.3.4 Flow cytometry staining protocol

For immunophenotyping, 200 µL of freshly obtained LN aspirate (mean cellularity typically >2,500 cells/µL as verified by hematology analyzer, frequently reaching higher concentrations in tumor-rich samples) was first incubated with BD Fc Block™ (BD Biosciences, Cat. 564220; 3 µL per sample, 10 minutes at room temperature) to prevent nonspecific Fc-mediated antibody binding. No washing step was performed, in accordance with the manufacturer’s recommendations. Subsequently, samples were incubated with pre-titrated monoclonal antibodies (2–5 µL per antibody, **Supplementary Table 1**) for 15 minutes at room temperature in the dark. Subsequently, 2 mL of BD Pharm Lyse Lysing Buffer (BD Biosciences, Cat. 55899) was added and samples were incubated for an additional 15 minutes in the dark at room temperature. Samples were then centrifuged (5 min, 1400 rpm), the supernatant was discarded, and the pellet was washed with 4 mL of BD Cell Wash buffer (BD Biosciences, Ref. 349524). After a second centrifugation (5 min, 1400 rpm), the pellet was resuspended in 200 µL Cell Wash buffer and immediately acquired on the flow cytometer.

### 2.4 Statistical analysis

Statistical analyses were performed using Statistica 13.0 (TIBCO Software, Palo Alto, CA, USA) and GraphPad Prism 8 (GraphPad Software, La Jolla, CA, USA). Continuous variables were summarized as mean ± standard deviation (SD) or median with interquartile range (Q1–Q3), depending on data distribution. Normality was assessed using the Shapiro–Wilk test. Comparisons between two independent groups were conducted using the Mann–Whitney U test for non-normally distributed variables. Results of antigens expression were reported as % and GMF. Correlations between continuous parameters were evaluated using non-parametric methods (Spearman’s rank correlation coefficient). Results are reported as rho (r). To account for multiple testing, correlations between PD-1/PD-L1/PD-L2 and non-classical checkpoints were additionally evaluated using the Benjamini–Hochberg false discovery rate (FDR) procedure. A p-value < 0.05 was considered statistically significant.

## 3 Results

### 3.1 Patient characteristics

The final study population consisted of 71 patients. All enrolled patients had a histological diagnosis of primary lung cancer with mediastinal LN metastases. The mean age of the study population was 67.6 ± 7.5 years, and the number of women and men in our study was similar (women n= 35, men n= 36). Patients were categorized according to tumor stage: stage I (n= 2, 2.8%), stage II (n= 3, 4.2%), stage III (n= 33, 46.5%), and stage IV (n= 33, 46.5%). However, after cytopathological confirmation of mediastinal LN metastases, the five cases initially recorded as stage I–II based on preliminary clinical staging were reclassified as stage III in accordance with TNM criteria (stage III n=38 and stage IV n=33). Among the enrolled patients, 33 had small cell lung cancer (SCLC) and 38 had NSCLC. Within the NSCLC subgroup, adenocarcinoma (AD) was the most common type (n= 22, 57.9%), followed by squamous cell carcinoma (SCC) (n= 9, 23.7%) and non-small cell lung cancer unspecified (NOS) (n= 6, 15.8%). Because of the limited number of cases within individual NSCLC subtypes, subtype-specific comparisons were not performed, and the NSCLC group was analyzed as a single category. A detailed summary of patient characteristics is presented in **Table 1**.

In flow cytometric analysis, immune molecules of tumor cells and basic T cell subpopulations were assessed as described in the Methods section. This paper presents results for the entire study population of lung cancer patients and then assesses differences both by histological tumor subtype (SCLC vs. NSCLC) and by tumor fraction in flow cytometric samples. Additional stratification based on tumor fraction in flow cytometry was performed as follows: low tumor fraction: ≤ 15% tumor cells in the sample (n = 16) and high tumor fraction: > 15% tumor cells in the sample (n = 55). Because the distribution of tumor−cell percentages in the cohort exhibited a clearly bimodal pattern, with one cluster of samples ranging from 0–14.9% and a second cluster ranging from 25.5–99.6% and no cases in the intermediate range (15–25%), patients were stratified into two groups using a 15% threshold. This cutoff therefore reflected a natural separation in the data rather than an arbitrary division. To assess the robustness of this stratification, sensitivity analyses using alternative thresholds (10% and 20%) were performed, yielding qualitatively similar results (**Supplementary Table 2**). To visualize the distribution of tumor-cell percentages, we generated a histogram, a kernel density estimation (KDE) plot, and a boxplot comparing samples with ≤15% and >15% tumor-cell content (**Supplementary Figure 2**). All three visualizations demonstrated a clearly bimodal distribution, with complete separation between the two groups and no samples falling between 15% and 25%. These plots illustrate that the 15% cutoff corresponds to a natural division in the data rather than an arbitrary threshold. Cytopathological evaluation confirmed metastatic involvement, and quantitative cytopathological tumor-cell percentages were available for 31 lymph-node samples. Flow-cytometric tumor fractions showed strong correlation with cytopathology (Spearman ρ = 0.82, p < 0.00001), with significantly higher cytopathological tumor content in samples >15% versus ≤15% by flow cytometry (median 80% vs 50%, p< 0.0132) and three cases in which flow cytometry underestimated tumor burden. These findings and the underlying distribution are illustrated in **Supplementary Figure 3**.

### 3.2 Cellular composition of LN aspirates and confirmation of malignant cell population

The cellular composition of LN aspirates showed a consistent overall pattern across patients, with tumor cells representing the dominant population and T lymphocytes constituting the major lymphocytes subset. Tumor cells accounted for a mean of 57.0% ± 35.0 of all events. Among leukocytes: lymphocytes represented 18.7% ± 24.1, of which T cells comprised 11.9% ± 15.3. Within the T-cell: CD4^+^ T cells were more abundant (8.0% ± 11.8) than CD8^+^ T cells (4.0% ± 4.5), with a mean CD4/CD8 ratio of 1.9 ± 1.4. Other leukocyte subsets were present at lower frequencies, including B cells (6.0% ± 12.7) and NK cells (1.5% ± 4.1). Neutrophils formed a variable but notable component of the aspirates (19.4% ± 20.7).

The tumor cells showed high expression of EpCAM and cytokeratin (EpCAM: 68.4% ± 25.8, 31062.1 GMF ± 51723.1 and cytokeratin: 57.2% ± 34.0, 44709 GMF ± 214695.9, respectively). These cells did not express the hematopoietic marker CD45. In flow cytometry, the tumor cells showed a characteristic pattern with high levels of SSC-A and fluorescence. Gating on the tumor cells allowed for subsequent analysis of immunological markers on the tumor cells, as well as separation of malignant cells from basic T cell subpopulations (**Figure 1**).

### 3.3 CD137/CD137L pathway expression

Analysis of the CD137/CD137L pathway revealed distinct expression patterns on tumor cells and lymphocytic subpopulations. CD137L was highly expressed on tumor cells (53.8% ± 20.2; 11845GMF ± 13925), whereas lymphocytic CD137L expression was lower overall (29.1% ± 21.3; 6417 GMF ± 4346). Within T lymphocytes, CD137L expression was higher (38.2% ± 31.5; 11499 GMF ± 14385), with CD4+ T cells exhibiting the highest levels (52.4% ± 39.3; 10766 GMF ± 9 478) compared to CD8+ T cells (41.1% ± 31.8; 7–865 GMF ± 6 792).

CD137 expression showed a somewhat different pattern. Tumor cells expressed CD137 at 30.7% ± 32.0 (1269 GMF ± 1 589), while lymphocytes had lower overall expression (24.2% ± 28.2; 2473 GMF ± 2994). Within T cells, CD137 expression was 27.2% ± 29.1 (3284 GMF ± 3043), with CD8+ T cells showing higher expression (44.6% ± 40.2; 9784 GMF ± 8607) than CD4+ T cells (13.5% ± 24.9; 2726 GMF ± 3127). All CD137 and CD137L expressions on tumor cells and T cell subpopulations are presented in **Table 2**.

**Table 2:** CD137/CD137L expression on tumor cells and lymphocyte subpopulations.

Marker	Population	% Positive cells (Mean ± SD)	GMF (Mean ± SD)
CD137L	Tumor cells	53.8 ± 20.2	11845± 13925
CD137L	Lymphocytes	29.1 ± 21.3	6417 ± 4346
CD137L	Lymphocys T cells	38.2 ± 31.5	11499 ± 14385
CD137L	CD4+ T cells	52.4 ± 39.3	10766 ± 9478
CD137L	CD8+ T cells	41.1 ± 31.8	7865 ± 6792
CD137	Tumor cells	30.7 ± 32.0	1269 ± 1589
CD137	Lymphocytes	24.2 ± 28.2	2473 ± 2994
CD137	Lymphocytes T cells	27.2 ± 29.1	3284 ± 3043
CD137	CD4+ T cells	13.5 ± 24.9	2726 ± 3127
CD137	CD8+ T cells	44.6 ± 40.2	9784 ± 8607

GMF, geometric mean fluorescence; SD, standard deviation.

CD137L was predominantly expressed on tumor cells and CD4+ T lymphocytes, with higher GMF in T cells compared to tumor cells. CD137 expression was more pronounced in CD8+ T lymphocytes than in tumor cells or CD4+ T cells, indicating a differential activation pattern within the TME. These patterns suggest activation of CD4+ T cells through CD137L and increased CD137 expression on cytotoxic CD8+ cells, consistent with their effector status.

### 3.4 CD200/CD200R pathway expression

Tumor cells exhibited higher CD200 expression compared to lymphocytes (28.9% ± 29.4; 2,348 GMF ± 5,709 vs. 6.7% ± 13.5; 3,328 GMF ± 3,108, respectively). Within T cell subsets, CD200 expression was low in both CD4+ T cells (9.3% ± 15.3; 1,739 GMF ± 6,280) and CD8+ T cells (14.8% ± 20.7; 1,395 GMF ± 2,563).

CD200R expression was relatively low on tumor cells (8.6% ± 13.5; 1,226 GMF ± 1,831), but higher in lymphocytes (17.3% ± 15.6; 3,328 GMF ± 3,108), particularly within T cells (19.3% ± 20.1; 4,190 GMF ± 4,828). Among T lymphocytes, CD8+ T cells showed markedly higher CD200R expression (49.0% ± 44.9; 16,941 GMF ± 17,561) compared to CD4+ T cells (9.5% ± 12.1; 2,772 GMF ± 2,158). The expression of CD200 and CD200R on tumor cells and T lymphocyte subpopulations is presented in **Table 3**.

**Table 3:** CD200/CD200R expression on tumor cells and lymphocyte subpopulations.

Marker	Population	% Positive cells (Mean ± SD)	GMF (Mean ± SD)
CD200	Tumor cells	28.9 ± 29.4	2,348 ± 5,709
CD200	Lymphocytes	6.7 ± 13.5	3,328 ± 3,108
CD200	Lymphocytes T cells	6.7 ± 13.9	1,447 ± 3,897
CD200	CD4+ T cells	9.3 ± 15.3	1,739 ± 6,280
CD200	CD8+ T cells	14.8 ± 20.7	1,395 ± 2,563
CD200R	Tumor cells	8.6 ± 13.5	1,226 ± 1,831
CD200R	Lymphocytes	17.3 ± 15.6	3,328 ± 3,108
CD200R	Lymphocytes T cells	19.3 ± 20.1	4,190 ± 4,828
CD200R	CD4+ T cells	9.5 ± 12.1	2,772 ± 2,158
CD200R	CD8+ T cells	49.0 ± 44.9	16,941 ± 17,561

GMF, geometric mean fluorescence; SD, standard deviation.

### 3.5 PD-1/PD-L1, PD-L2 pathway expression

PD-1 was detected on lymphocytes T, with 33.6% ± 16.0 of cells positive (3,568 GMF ± 2,420). Expression of PD-L1 and PD-L2 was observed on tumor cells, with PD-L1 present in 18.3% ± 21.5 of cells (2,730 GMF ± 15,897) and PD-L2 in 33.7% ± 25.7 of cells (860 GMF ± 683).

### 3.6 Interactions CD137/CD137L and CD200/CD200R pathways with PD-1/PD-L1/PD-L2 pathways

Correlation analysis examined associations between non-classical checkpoint pathways (CD137/CD137L and CD200/CD200R) with tumor burden and classical inhibitory checkpoints (PD-1, PD-L1, PD-L2) in LN aspirates. Only correlations reaching p < 0.05 were considered statistically significant. To improve statistical robustness and account for multiple testing (≈60 comparisons, n = 71), p-values were adjusted using the Benjamini–Hochberg false discovery rate (FDR) procedure. Because this analysis was exploratory in nature, both unadjusted and FDR-adjusted results are reported. Associations with FDR-adjusted q < 0.10 were considered statistically significant. As expected, most weak correlations (r ≈ 0.25–0.30) did not remain significant after FDR correction. However, the strongest associations—particularly CD137 on tumor cells vs. PD-L2 (r = 0.59), CD137 on lymphocytes vs. PD-1 (r = 0.42), and CD137 on CD4^+^ T cells vs. PD-1 (r = 0.50)—remained statistically significant after adjustment, indicating robustness to multiple-testing correction. For context, correlations in the range r = 0.27–0.42 correspond to r² values of approximately 7–18%, indicating small-to-moderate shared variance and supporting cautious interpretation of these associations.

#### 3.6.1 Tumor cells compartment

Within tumor-cell compartments, CD137 expression on tumor cells showed moderate positive correlations with PD-L1 and PD-L2 expression on tumor cells (r = 0.33 and r = 0.59, respectively). After FDR correction, only the CD137–PD-L2 association remained significant.CD137 expression on tumor cells also correlated with PD−1 on lymphocytes (r = 0.31). This correlation did not remain significant after FDR correction. Tumor-cell CD200R also correlated positively with PD-L1 and PD-L2 expression (r = 0.33 and r = 0.37, respectively). After FDR correction, only the CD200R–PD-L2 association remained significant. CD200 expression on tumor cells showed a positive correlation with PD-1 on lymphocytes (r = 0.27) and with PD-L2 on tumor cells (r= 0.33). Neither correlation remained significant after FDR correction.

#### 3.6.2 Lymphocyte populations

Within lymphocyte populations, CD137 expression on lymphocytes significantly positive correlated with PD-1 expression (r = 0.42),. This association remained significant after FDR correction. Similary, CD137 expression on lymphocytes T also significantly positive correlated with PD-1 expression (r = 0.42) also remaining significant after FDR adjustment. CD137L expression on T lymphocytes was negatively correlated with PD-L1 and PD-L2 expression (r = -0.27 and r = -0.38, respectively). Only the CD137L–PD-L2 correlation showed borderline significance after FDR correction.

#### 3.6.3 T lymphocyte subpopulations

In CD4^+^ T cell subsets, CD137L expression correlated negatively with PD-L1 and PD-L2 on tumor cells (r = -0.26 and r = -0.39, respectively, *p* < 0.05). The CD137L–PD-L2 association remained significant after FDR correction. CD137 expression on CD4^+^ T cells correlated positively with PD-1 expression on lymphocytes (r = 0.50). This correlation remained significant after FDR adjustment and represents a moderate effect size (r² ≈ 25%).

**Figure 2:**
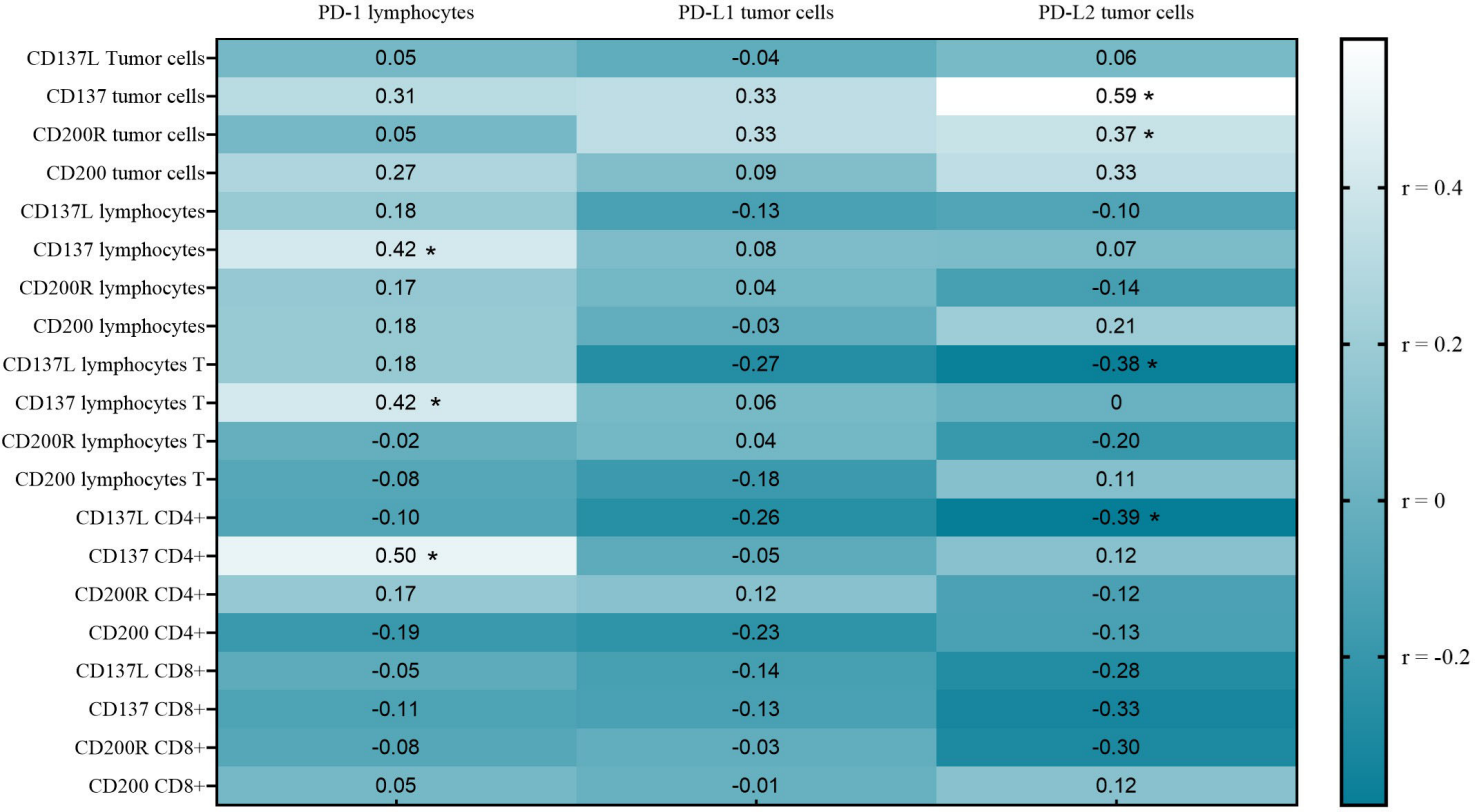
Heatmap illustrating Spearman correlation coefficients (r) between classical immune checkpoints (PD-1, PD-L1, PD-L2) and non-classical checkpoints (CD137, CD137L, CD200, CD200R) across tumor cells and lymphocyte subsets in metastatic lymph node aspirates. Correlation coefficients are displayed within each cell. Asterisks (*) indicate correlations that remained statistically significant after Benjamini–Hochberg false discovery rate (FDR) correction (q < 0.10).

Negative correlations were observed between CD137L CD8^+^ T cells and PD-L2 tumor cells (r = -0.28) and between CD137 CD8+T cells and PD-L2 tumor cells (r = -0.33), and CD200R CD8^+^ T cells and PD-L2 (r = -0.30). These correlations did not remain significant after FDR correction. A full list of correlation coefficients, is provided on heatmaps (**Figure 2**), with correlations that remained statistically significant after FDR correction indicated by an asterisk (*).

### 3.7 Differences in the CD137/CD137L, CD200/200L and PD-1/PD-L1, L2 pathway expression: depending on the type of cancer

Comparative analysis between NSCLC (n = 38) and SCLC (n = 33) revealed several statistically significant differences in the expression of selected checkpoint molecules within LN aspirates.

CD137 expression on tumor cells was higher in NSCLC both in terms of percentage of positive cells (28.6% vs. 4.3%; p= 0.0007) and GMF (1154 vs. 433; p< 0.0001).

No significant differences were found in CD137 or CD137L expression on lymphocytes or their T-cell subsets.

**Figure 3:**
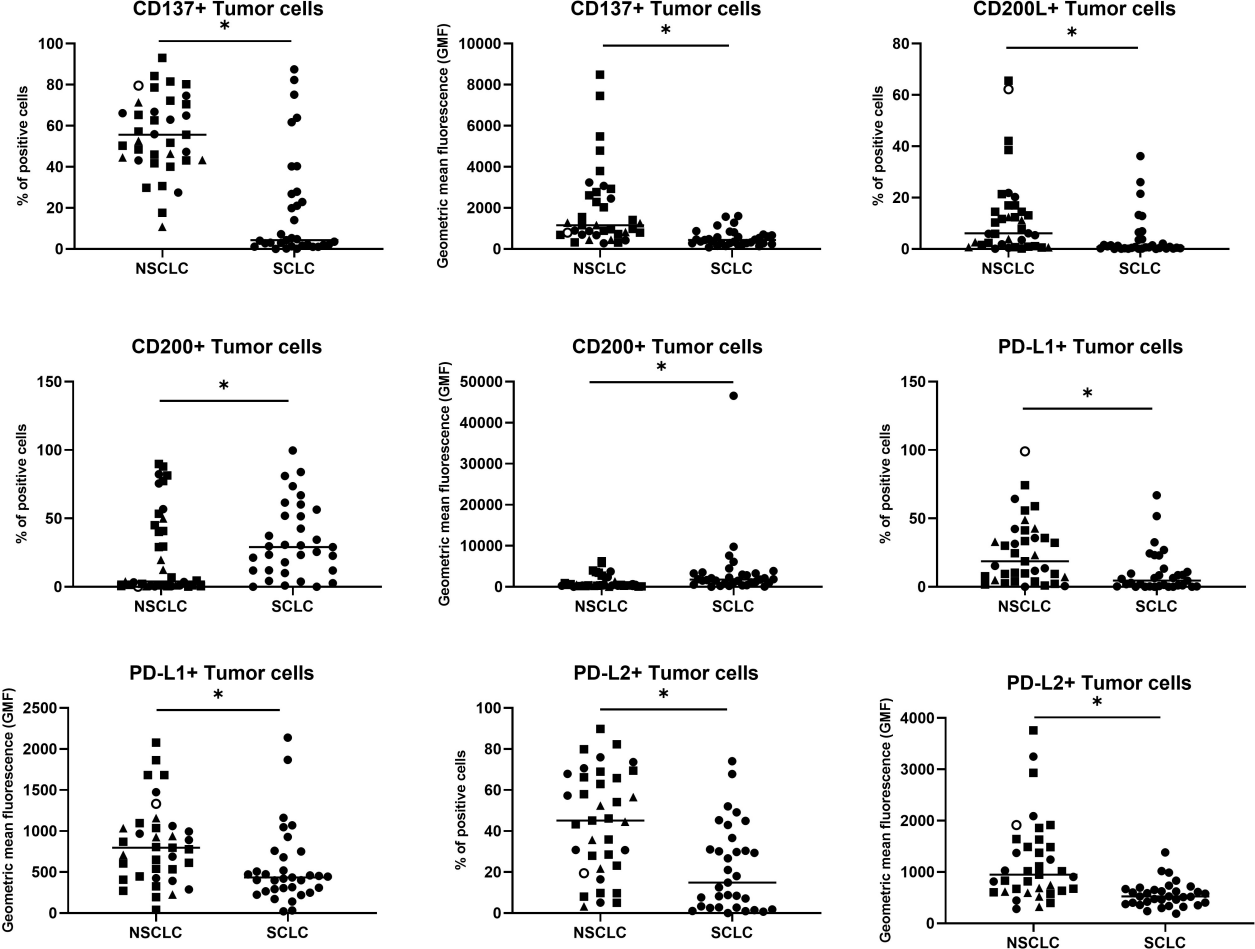
Statistically significant differences in the expression ratios of CD137/CD137L, CD200/200L, and PD-1/PD-L1, L2 in the cell subpopulations in lymph node aspirates (LNs) between patients with non-small cell lung cancer (NSCLC) and small cell lung cancer (SCLC). Data are presented as median with individual data points.Within the NSCLC group, individual samples are annotated according to histological subtype: adenocarcinoma (◼), squamous cell carcinoma (•), large cell carcinoma (○), and not otherwise specified (▲). *Statistical significance: p < 0.05.

Within the CD200/CD200R pathway, tumor-cell CD200R expression was significantly higher in NSCLC than in SCLC (6.1% vs. 0.8%; p = 0.0003). In contrast, tumor-cell CD200 expression was significantly higher in SCLC than in NSCLC, both in the percentage of CD200-positive cells (29.1% vs. 3.9%; p = 0.0495) and in GMF (1757 vs. 433; p = 0.0034). CD200/CD200R expression on lymphocytes and T-cell subsets did not differ significantly between tumor types. Classical checkpoint expressions PD-1/PD-L1 and PD-L2 revealed the most prominent differences between-groups. Both PD-L1 and PD-L2 were markedly higher on tumor cells in NSCLC compared with SCLC, in terms of percentage (PD-L1: 18.7% vs. 4.5%; PD-L2: 45.1% vs. 14.9%) and GMF (PD-L1: 797 vs. 435; PD-L2: 948 vs. 525), with statistical significance (p≤ 0.0045). PD-1 expression on lymphocytes did not differ between NSCLC and SCLC groups. Statistically significant results are presented in **Figure 3**. All results with the proportion of CD137/CD137L, CD200/200R and PD-1/PD-L1, L2 expression on cell subpopulation (tumor cells and lymphocytes) in LNs between patients with NSCLC and SCLC are presented in **Supplementary Table 3**.

### 3.8 Association of tumor fraction with CD137/CD137L, CD200/CD200R, and PD-1/PD-L1/PD-L2 pathway expression

As the amount of tumor cells within the metastatic LNs may influence local immune interactions in TME, samples were stratified into low (≤15%) and high (>15%) tumor fraction groups for further analysis. Analysis revealed distinct differences limited to selected checkpoints, mainly within tumor cells. Tumor CD137 expression was markedly lower in high–tumor-fraction samples, both in % positivity (12.7% vs. 47.7%, p = 0.0178) and in GMF (575 vs. 2028, p < 0.0001). Tumor CD137L did not differ between groups. Similarly, CD137/CD137L expression on lymphocytes, including CD4+ and CD8+ subsets, showed no significant differences.

Tumor CD200R expression was significantly lower in high–tumor-fraction samples (1.4% vs. 11.2%, p = 0.0459), whereas GMF did not differ. Other CD200/CD200R parameters on lymphocytes and T-cell subsets did not show significant differences between groups.

**Figure 4:**
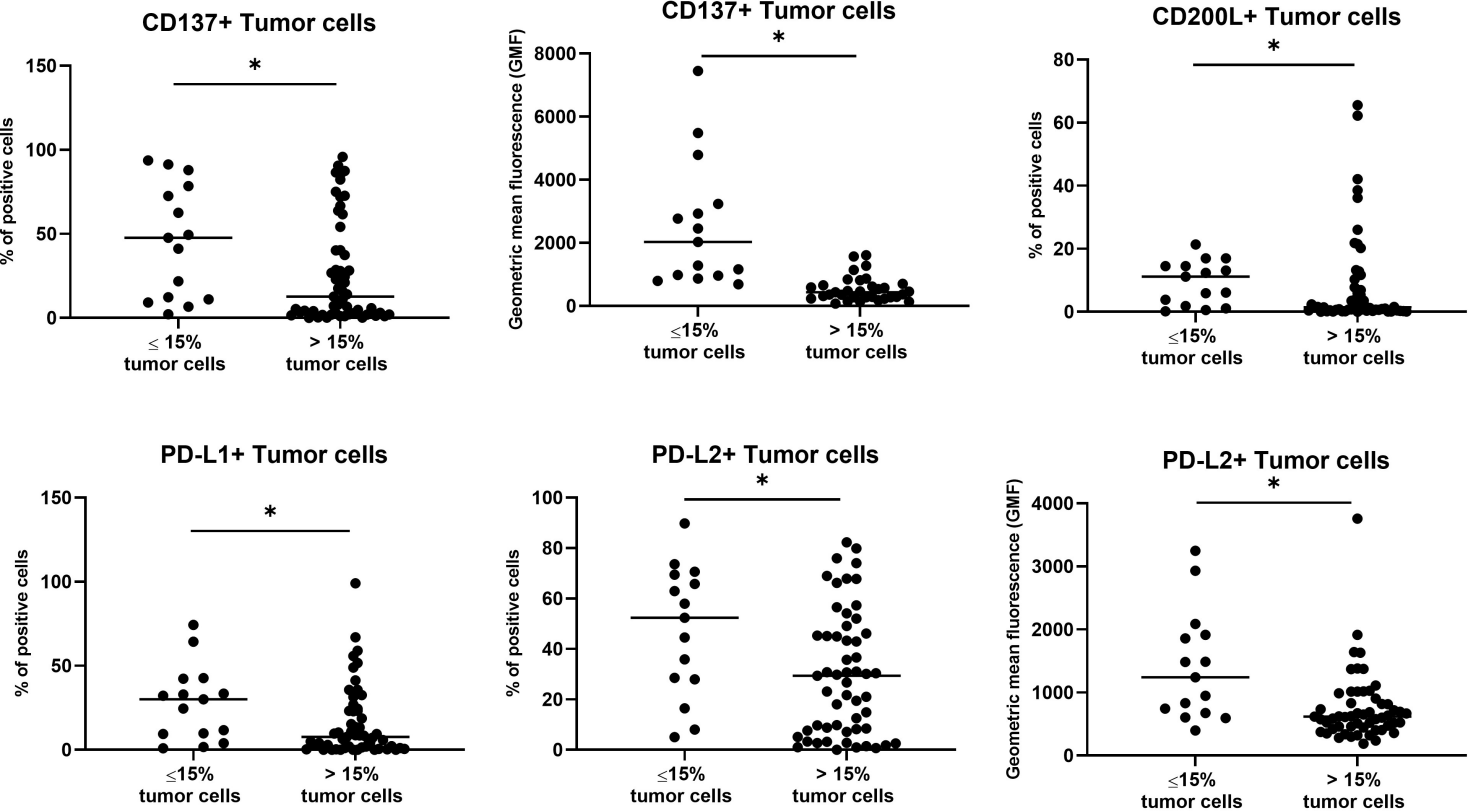
Statistically significant differences in the expression ratio of CD137/CD137L, CD200/200L, and PD-1/PD-L1, L2 in lymph node (LN) aspirates between patients with low and high tumor fraction in the flow cytometry sample. Data are shown as individual values with the median indicated. Tumor fraction stratification was defined as low (≤15% tumor cells) and high (>15% tumor cells). *Statistical significance: p < 0.05.

Tumor PD-L1 positivity was reduced in the high-tumor-fraction group (7.7% vs. 30.0%, p = 0.0193). Tumor PD-L2 showed even more pronounced differences, both in % positivity (29.4% vs. 52.3%, p = 0.0275) and GMF (617 vs. 1242, p = 0.0011). PD-1 expression on lymphocytes did not differ significantly between groups. Statistically significant results are presented in **Figure 4**. All results with the proportion of CD137/CD137L, CD200/200L and PD-1/PD-L1, L2 expression on cell subpopulation (tumor cells and lymphocytes) in LNs between patients with low and with high tumor fraction are presented in **Supplementary Table 4**. All patients in the low tumor-fraction group (≤15%) had NSCLC (n = 16), whereas the high tumor-fraction group (>15%) included both NSCLC (n = 22) and SCLC (n = 33). Tumor-fraction stratification was performed independently of histological subtype, and no subtype-specific comparisons were conducted within these groups.

## 4 Discussion

### 4.1 Metastatic LNs

In this study, we demonstrate that the non-classical immune checkpoints: CD137/CD137L and CD200/CD200R are expressed in LNs of lung cancer patients and show meaningful differences on various TME cell populations. By applying multiparameter flow cytometry to EBUS/TBNA aspirates, we characterize the local immune TME at the site of lung cancer metastases. Our data confirm that EBUS/TBNA-derived LN aspirates represent a cell-rich material suitable for immune profiling. Cancer cells constituted the predominant population in metastatic LNs, distinguished by EpCAM/cytokeratin expression, light scatter characteristics, and CD45 negativity, with T cells constituting the major lymphocyte subset. This immunophenotyping enabled subsequent assessment of the non-classical immune checkpoints CD137/CD137L and CD200/CD200R. Metastatic LNs represent a relevant site for studying interactions between tumor cells and local immune populations (28, 29). Previous studies, including ours, that used flow cytometry to detect EBUS/TBNA have mainly focused on basic leukocyte subpopulations or classical immune checkpoints, demonstrating the feasibility and diagnostic value of this approach (22, 25, 26, 29, 30). Evaluation of non-classical immune checkpoint pathways in metastatic LNs has been scarce, and our study expands this area by providing detailed characterization of CD137/CD137L and CD200/CD200R expression within this anatomical niche.

### 4.2 CD137/CD137L pathway

Studies confirm that the CD137/CD137L axis is a context-dependent regulator of antitumor immunity (31). In our LN aspirates, CD137L predominated on tumor cells (mean expression 53.8%), whereas CD137 was present on CD8^+^ T cells (mean expression 44.6%). CD137L expression in tumor cells has previously been described in tissues from patients with NSCLC using immunohistochemistry. Experimental studies suggest that CD137L can mediate reverse signaling in tumor cells, influencing proliferation and apoptosis in NSCLC models (32). However, the biological consequences of this signaling appear to be context-dependent and may vary across tumor types. Previous studies have also reported CD137L expression in several solid tumors, supporting the biological relevance of this pathway within the tumor microenvironment (33–35).

In addition to the expression of CD137L associated with tumor cells, we also unexpectedly detected CD137L on a subset of CD4^+^ T cells in LN aspirates. As CD137L is classically described on antigen-presenting cells, its detection on CD4^+^ T cells requires cautious interpretation. CD137L expression on CD4+ T cells is not classically described in cancer and may seem unusual. To date, only one study has directly demonstrated CD137L expression on CD4^+^ T cells, specifically within the regulatory T−cell compartment in an autoimmune setting (36). Beyond this report, robust evidence supporting CD137L expression on conventional CD4^+^ T cells in humans is scarce. Therefore, the detection of CD137L on CD4^+^ T cells in metastatic lymph node aspirates should be regarded as an exploratory observation that requires further validation.In contrast, CD137 expression on CD8+ T cells is well documented and considered a marker of functional activation of cytotoxic lymphocytes in the TME. High expression of CD137 on CD8+ T cells was demonstrated in our study (mean expression 44.6%). In CD8^+^ T cells, CD137 expression is usually higher and persistent, while on CD4+ it is less frequent and often less intense (37), which is consistent with our study. Previous studies indicate that CD137 expression identifies activated CD8^+^ T cells and may mark tumor-reactive lymphocyte populations within the tumor microenvironment (38–42).

In summary, our data indicates that metastatic LNs are a site where components of the CD137/CD137L pathway are prominently expressed on tumor cells and CD8^+^ T cells.This observation suggests that the CD137/CD137L axis may contribute to local immune regulation within metastatic LNs.

### 4.3 CD200/CD200R pathway

Analysis of the CD200/CD200R pathway demonstrated distinct expression patterns between tumor cells and lymphocytic subpopulations. In our study, tumor cells were the primary source of CD200 expression, while CD200R expression was relatively low on tumor cells and significantly higher in the lymphocyte population, particularly among cytotoxic CD8+ lymphocytes. Our results are consistent with previous reports indicating that CD200 is frequently expressed on tumor cells, stromal cells, or endothelial cells, whereas CD200R is expressed primarily on immune cells, including T lymphocytes and myeloid cells (18). The presence of CD200 on tumor cells may promote local immunosuppression through interaction with CD200R on effector cells (43, 44), a mechanism that has been associated with inhibition of antitumor immune responses in experimental models (43, 45). However, increasing evidence suggests that the role of the CD200/CD200R axis in cancer is not unequivocally suppressive and may depend on the biological context, tumor type, and microenvironmental composition. In lung cancer, expression of the CD200R correlates with both the presence of T lymphocytes and a more aggressive tumor phenotype, highlighting its complex biological role. Yoshimura K. et al. (18), immunohistochemically assessed CD200 and CD200R expression in NSCLC tumor cells and stroma (including the TME) and confirmed its association with clinicopathological features, including survival. CD200 receptor expression was high in tumor or stromal cells, correlated with higher TIL counts, and associated with poorer survival. In our study, CD200R expression on tumor cells was significantly lower (average 8.6%) compared to CD200 expression (average 28.9%). In this context, high expression of CD200R on CD8+ lymphocytes may reflect the existence of a subpopulation of cytotoxic lymphocytes present in metastatic LNs, potentially involved in an active immune response to tumor cells. This phenotype may represent an effector or regulated T-cell population rather than a purely dysfunctional state. Recent multi-tumor analyses using flow cytometry and single-cell transcriptomics confirm that CD200R is expressed not only on myeloid cells but also on CD8^+^ T cells within the tumor microenvironment (46, 47). This confirms that high CD200R expression on cytotoxic T cells may represent a prominent immunoregulatory feature within the tumor microenvironment of solid tumors. Yao Z. et al. (48) reviewed CD8+ T cell phenotypes in solid tumors, discussing multiple inhibitory receptors, including CD200R1 on CD8+ T cells. Chronic antigenic stimulation (as in the TME) has been shown to promote CD200R1 induction on CD8+ T cells, and signaling through this receptor can limit their effector activity, making CD200R1 a potential target for reversing cytotoxic T cell dysfunction. Tøndell et al. (49) findings indicate that in NSCLC, the CD200/CD200R axis is an integral component of a complex network of checkpoints in the tumor microenvironment, encompassing interactions between tumor cells, macrophages, and T cells. The authors emphasize that the presence of CD200R on immune cells does not necessarily reflect an effective antitumor response, but rather a context-dependent modulation of immune activity. Our data are consistent with this model and indicate that analogous regulatory mechanisms may also occur in the LNs microenviroment.

### 4.4 PD-1/PD-L1, PD-L2 pathways and relationship with non-classical pathways

Our study demonstrated frequent PD-1 expression on lymphocytes (mean 33.6%) and detectable PD-L1 and PD-L2 expression on tumor cells, with higher PD-L2 than PD-L1 (mean 33.7% vs. 18.3%). These findings confirm activation of the PD-1/PD-L1/PD-L2 axis in metastatic LNs. Interestingly, although a higher percentage of tumor cells expressed PD-L2 compared with PD-L1, the GMF of PD-L2 was significantly lower, suggesting that PD-L2 expression is less intense in individual cells. These results are consistent with previously reported patterns in lung cancer and demonstrate the differential contribution of PD-L1 and PD-L2 to the TME (50). Importantly, studies using EBUS/TBNA-derived LNs aspirates, including our work, demonstrated PD-L1 expression on cancer stem cells (30), and support the feasibility of assessing PD-1/PD-L1 pathways in LN metastases as a reflection of the TME (24).

Because the correlation analysis was exploratory and most effect sizes were modest (r ≈ 0.27–0.42; r² ≈ 7–18%), all associations require cautious interpretation and should not be viewed as mechanistic. Two correlations showed stronger effect sizes (CD137 tumor cells–PD-L2, r = 0.59; CD137 CD4^+^–PD-1, r = 0.50), but these also reflect co-expression patterns rather than functional interactions. Correlation analyses suggest that classical checkpoints do not function independently of non-classical pathways in metastatic LNs. Vathiotis et al. reported that CD200 expression correlates with PD-L1 in NSCLC tissues (13). In contrast, in our cohort CD200 on tumor cells did not show significant correlations with PD-L1 or PD-L2 after FDR correction. These differences may reflect biological heterogeneity between primary tumors and metastatic LNs, as well as methodological differences between immunohistochemistry-based and flow-cytometry-based analyses. Among tumor-cell pathways, only CD137–PD-L2 and CD200R–PD-L2 remained significant after FDR correction, representing modest but reproducible associations. These findings suggest that PD-L2 may interact more broadly with non-classical checkpoints than PD-L1, consistent with reports describing a distinct biological role for PD-L2 in NSCLC (50, 51).

Specifically, PD-L2 expression on tumor cells was positively associated with CD137 on tumor cells after FDR correction. Other tumor-cell correlations involving CD137 or CD200 did not withstand FDR correction and should be considered hypothesis-generating. To the best of our knowledge, no studies to date have systematically explored the interdependence between the CD137/CD137L axis and the PD-1/PD-L1/PD-L2 pathways in lung cancer tissues. Existing literature focuses largely on functional activation of CD137 in T and NK cells or on the effects of agonistic anti-CD137 antibodies, as well as on therapeutic strategies combining CD137 with PD-1/PD-L1 blockade (52–54), but direct analyses of co-expression patterns within tumor cells or metastatic LNs are lacking. Therefore, the correlations identified in our study should be interpreted as exploratory evidence of potential crosstalk rather than mechanistic interactions. Within lymphocyte populations, the strongest association in our dataset was the moderate positive correlation between CD137 expression and PD-1 (r = 0.42; r² ≈ 18%), reflecting a subset of T cells co-expressing an activation-associated costimulatory receptor and an inhibitory receptor. This likely reflects a subset of chronically stimulated, potentially tumor-reactive T cells, but without functional assays no conclusions regarding exhaustion can be drawn (55). We did not perform functional assays (e.g., cytokine production, proliferation, cytotoxicity), nor did we assess additional inhibitory receptors such as TIM-3, LAG-3 or TIGIT, which are typically required to define exhausted T cells; therefore, any inference about functional impairment would be speculative. However no such correlation was observed for CD200R. Prior studies have shown that CD200R and PD-1 co-expression may reflect chronic antigenic stimulation rather than terminal dysfunction (56).

Two additional correlations remained significant after FDR correction: CD137L CD4^+^–PD-L2 and CD137L T-lymphocytes–PD-L2. These negative associations may indicate that activation-related signals (CD137/CD137L) coexist with lower PD-L2 expression in certain metastatic niches, although this observation remains exploratory and has not been confirmed by other groups.Several negative correlations with PD−L2 were observed in CD8^+^ T cells, but none remained significant after FDR correction. Although PD-L2–rich microenvironments have been associated with stronger CD8^+^ T-cell dysfunction in prior studies (57–59) our data cannot confirm this relationship without functional validation. Overall, metastatic LNs appear to contain a complex but measurable network of classical and non-classical immune checkpoints. The seven correlations that remained significant after FDR correction highlight potential points of interaction, but their biological relevance requires further investigation. As the literature offers few directly comparable analyses—particularly those using multiparametric flow cytometry of EBUS/TBNA samples—contextual interpretation remains limited. Nevertheless, our findings provide exploratory insight into checkpoint interactions within the TME and underscore the need for future studies integrating functional assays and clinical outcomes.

### 4.5 Impact of tumor type and tumor cell burden on immune checkpoint expression in metastatic LNs

In our study, we confirmed that the histological type of lung cancer influences the expression of selected checkpoints primarily on tumor cells in metastatic LNs, whereas the profile of lymphocyte checkpoint molecules remains unchanged. It is known that NSCLC and SCLC histological subtypes differ significantly in terms of immunogenicity, mutational burden, stromal composition, and response to immunotherapy, with NSCLC generally exhibiting stronger immune infiltrates than small cell lung cancer SCLC (60–62).

Higher expression of CD137 on tumor cells in NSCLC may reflect a greater potential for costimulatory interactions within the local TME compared to SCLC. However, the literature on CD137 expression on tumor cells is limited. The functional role of CD137 in tumor cells remains largely unknown, and studies that identify CD137 as a marker of activated T/NK cells predominate (6, 38). Thus, our observation of histology-dependent variation in tumor-cell CD137 adds new information on this pathway within the nodal tumor microenvironment. The differential expression of CD200 and CD200R across NSCLC and SCLC further supports the concept that these tumors adopt distinct immunoregulatory strategies. Elevated CD200R expression on tumor cells in NSCLC may indicate adaptive tumor responses to immune activity within the TME. Studies have shown that the CD200/CD200R axis plays a key role in regulating the immune response, although CD200R is mainly expressed on myeloid cells and some lymphocytes, acting as an inhibitory receptor regulating the activation of effector cells of the immune system (63). An interesting observation in our study was higher expression of CD200 on SCLC tumor cells compared with NSCLC. The dominant expression of CD200 in SCLC may promote a stronger immunosuppressive signal by interacting with cells expressing CD200R, which is consistent with the more aggressive biology of this tumor subtype. The authors also indicate that increased expression of CD200 in tumors is one of the mechanisms of immune escape and a potential target for new immunotherapy strategies (43). Coles et al. showed that high expression of CD200 on tumor cells leads to strong immunosuppression by inhibiting the function of NK cells and T lymphocytes via CD200R-positive cells (64).

The most pronounced differences were observed in the classical PD-1/PD-L1/PD-L2 checkpoint pathway, where higher expression of PD-L1 and PD-L2 on tumor cells in NSCLC suggests a greater ability of this subtype to modulatelocal immune response in metastatic LNs. This finding is consistent with previous reports showing that NSCLC is generally more immunogenic than SCLC and displays higher PD-L1/PD-L2 expression, which may partly explain its greater sensitivity to immune checkpoint inhibitor therapy (65–68). According to literature reports, PD-1 expression on lymphocytes demonstrates less ability to differentiate between histological types and may reflect the general state of immune activation rather than tumor-specific characteristics (69–71). There are no studies directly comparing PD-1 expression on infiltrating lymphocytes between NSCLC and SCLC in material obtained by EBUS/TBNA.

In addition to differences related to histological type, our study highlighted tumor cell burden within metastatic LNs as a potential factor influencing local immune interactions. Metastatic LNs are characterized by variable percentages of tumor cells and immune infiltrate, and increasing tumor mass may influence changes in immunoregulatory axes (28). In our cohort, the observed differences concerned the expression of checkpoints on tumor cells, whereas the checkpoint profile of lymphocytes remained stable across tumor-fraction groups. In samples with high tumor fraction, significantly lower expression of CD137 and reduced expression of CD200R on tumor cells were observed. Studies on cancer progression have shown that the increasing tumor mass in the lymph node leads to remodeling of the local microenvironment, shifting it from an immunologically active phase towards a more tolerogenic one (72, 73).

The most substantial differences were observed in checkpoints associated with the classical PD-1 pathway. Reduced expression of PD-L1 and PD-L2 was observed on tumor cells in samples with a high tumor fraction. There are no studies in the literature confirming this observation, but previous analyses have shown that PD-L1 and PD-L2 expression on tumor cells is highly heterogeneous and may be dynamically regulated during tumor progression, varying depending on immune infiltration and microenvironmental remodeling, rather than remaining stable across disease stages (74–77).

The simultaneous lack of differences in checkpoint expression on lymphocytes between the low and high tumor fraction groups suggests that tumor burden in metastatic LNs primarily modulates checkpoint expression on tumor cells rather than lymphocyte populations.

### 4.6 Limitations

This study has several limitations that should be considered. First, the analysis was based on LN aspirates obtained by EBUS/TBNA, which, although well suited for multiparametric flow cytometry, do not preserve tissue architecture. A further limitation of this study is the lack of non-metastatic lymph node controls, as EBUS/TBNA sampling in routine clinical practice is performed only on nodes with radiological or endosonographic suspicion of malignancy. Therefore, the presented results reflect the immune landscape of metastatic LNs rather than baseline lymph node immunophenotypes. Future prospective studies including paired metastatic and non-metastatic LNs would be valuable to better distinguish tumor-specific immune modulation from physiological nodal immune biology. Furthermore in samples with low tumor burden, the reduced percentage of malignant cells may partially reflect sampling from peritumoral areas or heterogeneously involved LNs rather than a true biological absence of malignant cells, which should be considered when interpreting analyses based on tumor fraction. In addition, stratification using a 15% tumor-cell percentage threshold represents a methodological constraint. Although this cutoff was not derived from clinical outcome data, it corresponded to a naturally bimodal distribution observed in our cohort, with no samples falling between 15% and 25%, suggesting separation of two distinct clusters rather than arbitrary division of a continuous variable, Nevertheless, tumor-cell percentages in EBUS/TBNA aspirates may be influenced by sampling variability and mechanical loss of epithelial cells inherent to aspiration procedures. Sensitivity analyses using alternative thresholds (10% and 20%) yielded consistent trends, supporting the robustness of the observed associations. Future studies integrating quantitative cytopathological assessment may allow more precise calibration of tumor cell–based stratification. Direct quantitative correlation between cytopathological tumor estimates and flow cytometric tumor percentages was not performed in this cohort and would represent a valuable direction for future validation studies. Third, although flow cytometry provides high-resolution single-cell immunophenotyping, the study did not include orthogonal validation (e.g., immunohistochemistry) of non-classical checkpoint expression. These markers are not routinely assessed in diagnostic IHC panels for EBUS/TBNA-derived material, but complementary validation would strengthen future translational studies. Moreover the study was cross-sectional and did not include functional testing or longitudinal sampling. Therefore, the functional consequences of CD137/CD137L and CD200/CD200R expression were not examined. Additionally, the unexpected detection of CD137L on CD4^+^ T cells was not validated using alternative antibody clones or transcriptomic confirmation and should therefore be considered exploratory. Despite these limitations, EBUS/TBNA-derived LNs provide a rapid, minimally invasive, and cell-rich material for immune profiling, enabling detailed characterization of tumor–immune interactions in metastatic LNs. Our findings support the utility of flow cytometry-based analysis of LN aspirates that complements standard histopathological evaluation in lung cancer diagnostics. Clinical outcome data (treatment response, survival) were not available for this diagnostic cohort, and future prospective studies integrating checkpoint expression with clinical endpoints will be essential to assess their therapeutic relevance.

## 5 Conclusion

In this study, we demonstrated that nonclassical immune checkpoints CD137/CD137L and CD200/CD200R are expressed within metastatic LNs of lung cancer patients and show distinct distribution patterns across tumor and immune cell populations. They exhibit differential expression among tumor and immune cell populations within the TME and are associated with components of the classical PD-1/PD-L1/PD-L2 axis. Tumor cells constituted the major compartment expressing CD137L, CD200, and ligands of the PD-1 pathway, whereas lymphocyte subsets showed a relatively stable checkpoint profile across different histological subtypes and tumor cell burdens.

Importantly, our results indicate that histological subtype influences the expression of several immune checkpoints primarily at the tumor-cell level in metastatic LNs, whereas lymphocyte checkpoint profiles remain comparatively stable.

Stratification according to tumor-cell fraction further revealed differences in checkpoint expression on tumor cells between low and high tumor-fraction groups, suggesting that the extent of tumor involvement in LNs is associated with variation in checkpoint expression patterns.

Our findings provide new insights into the immune checkpoint landscape of metastatic LNs in lung cancer, highlighting the combined influence of tumor histology and tumor-cell burden on checkpoint expression patterns. Furthermore, t multiparametric flow cytometric analysis of LN aspirates enables detailed characterization of these interactions within metastatic LNs and may serve as a starting point for future studies exploring immunoregulatory mechanisms in lung cancer.

Furthermore, multiparametric flow cytometric analysis of LN aspirates enables detailed characterization of immune checkpoint expression within metastatic LNs and may serve as a starting point for future studies exploring immunoregulatory mechanisms in lung cancer.

## Data Availability

The original contributions presented in the study are included in the article/**Supplementary Material**. Further inquiries can be directed to the corresponding author.

## Supplementary material

Supplementary Figure 1 Representative histograms illustrating the gating strategy and fluorescence-minus-one (FMO) control–based assessment of immune checkpoint expression in lymph node aspirates. **(A)** FMO controls and corresponding marker-stained histograms for tumor cells, shown for selected checkpoint molecules (CD137, CD137L, CD200, CD200R, PD-L1, PD-L2). Gates for positive populations were defined using FMO-derived negative boundaries. **(B)** Representative FMO and marker-stained histograms for total lymphocytes, shown for selected checkpoint molecules (CD137, CD137L, CD200, CD200R, PD-1).

Supplementary Figure 2 Distribution of tumor−cell percentages in EBUS−TBNA aspirates. **(A)** Histogram and **(B)** kernel density estimation (KDE) curves showing the distribution of tumor−cell percentages in samples classified as low−tumor (≤15%) and high−tumor (>15%). The two groups form completely non−overlapping distributions, with all low−tumor samples ranging from 0–14.9% and all high−tumor samples ranging from 25.5–99.6%. No values were observed in the intermediate range (15–25%), indicating a naturally bimodal distribution. **(C)** Boxplot comparing the two groups, demonstrating complete non−overlap of ranges and markedly different medians. Together, these visualizations support the use of the 15% cutoff as a data−driven and biologically meaningful stratification.

Supplementary Figure 3 **(A)** Scatterplot showing the relationship between flow cytometry–derived tumor cell percentage (FC %) and cytopathological tumor content (ICH %) in the subset of 31 lymph node (LN) samples with quantitative cell block assessment. A strong correlation was observed between the two modalities (Spearman ρ = 0.82, p < 0.00001), with three cases in which flow cytometry underestimated tumor content. Colors indicate samples with low (≤15%) and high (>15%) tumor cell content by flow cytometry. **(B)** Boxplot comparing ICH % tumor cell content between samples classified as ≤15% and >15% tumor cells by flow cytometry, using the same color scheme as in panel A. Samples with >15% tumor cells by flow cytometry demonstrated significantly higher cytopathological tumor fractions (median 80% vs 50%, Mann–Whitney p = 0.0132).
