# Supplementary figures and images for: Non-classical immune checkpoint CD137/CD137L and CD200/CD200R expressions are regulated by the tumor immune microenvironment in lymph node aspirates from lung cancer patients
Source: Front Immunol. 2026 May 26;17:1766726. doi: 10.3389/fimmu.2026.1766726 (PMC13246615; doi:10.3389/fimmu.2026.1766726)

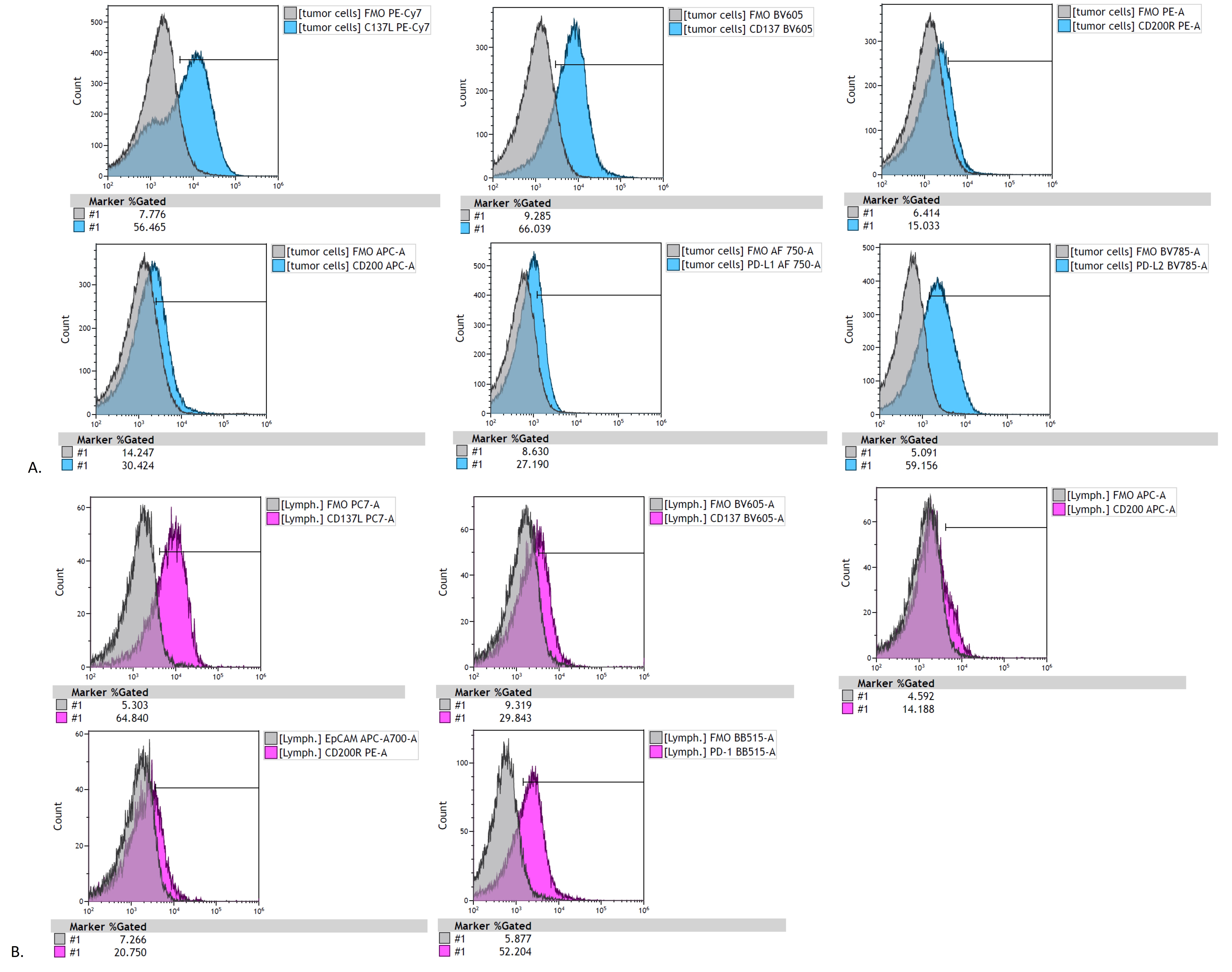

Supplement: Supplementary Figure 1 — Representative histograms illustrating the gating strategy and fluorescence-minus-one (FMO) control–based assessment of immune checkpoint expression in lymph node aspirates. (A) FMO controls and corresponding marker-stained histograms for tumor cells, shown for selected checkpoint molecules (CD137, CD137L, CD200, CD200R, PD-L1, PD-L2). Gates for positive populations were defined using FMO-derived negative boundaries. (B) Representative FMO and marker-stained histograms for total lymphocytes, shown for selected checkpoint molecules (CD137, CD137L, CD200, CD200R, PD-1). [file Image1.jpeg]

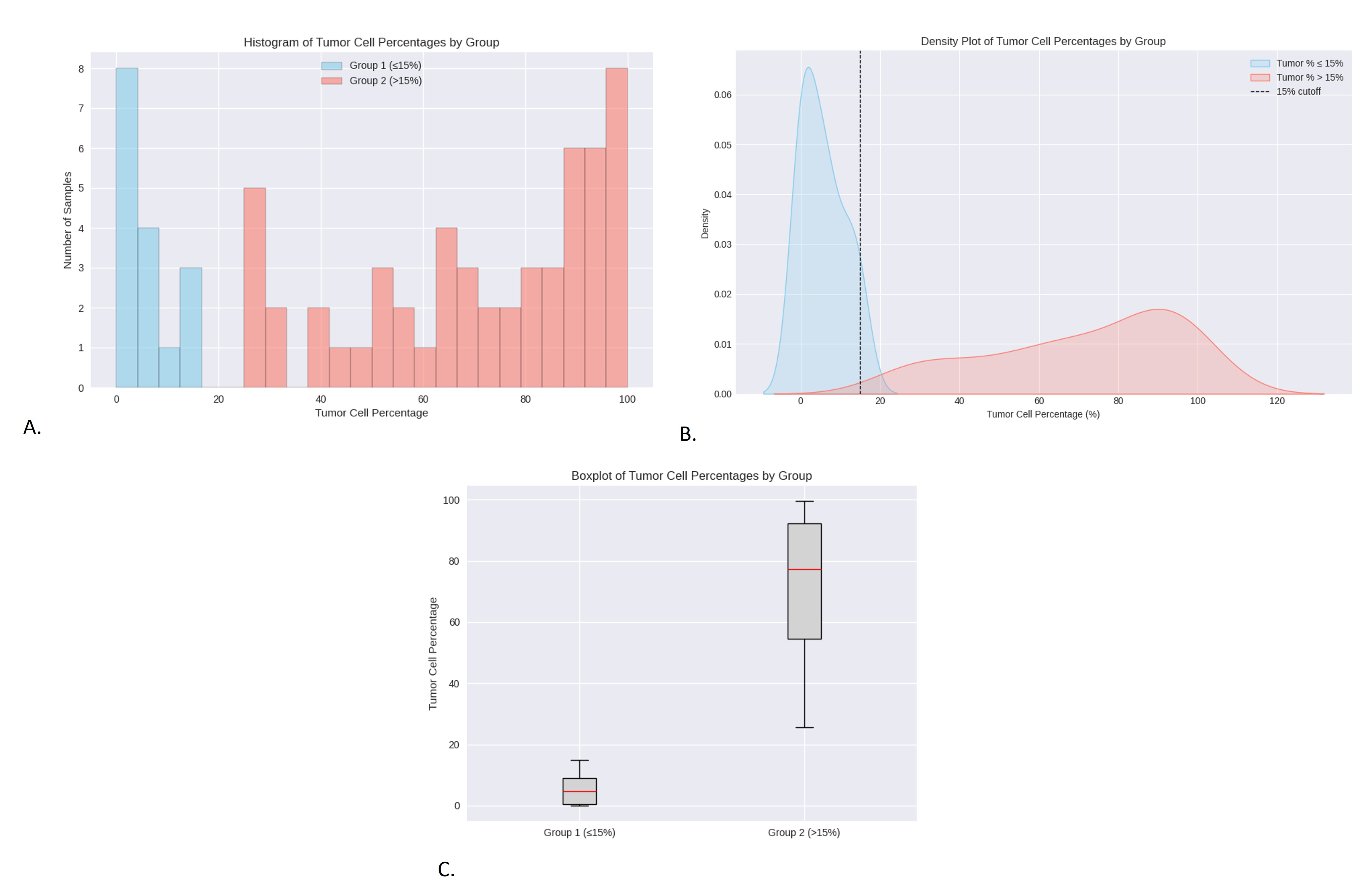

Supplement: Supplementary Figure 2 — Distribution of tumor−cell percentages in EBUS−TBNA aspirates. (A) Histogram and (B) kernel density estimation (KDE) curves showing the distribution of tumor−cell percentages in samples classified as low−tumor (≤15%) and high−tumor (>15%). The two groups form completely non−overlapping distributions, with all low−tumor samples ranging from 0–14.9% and all high−tumor samples ranging from 25.5–99.6%. No values were observed in the intermediate range (15–25%), indicating a naturally bimodal distribution. (C) Boxplot comparing the two groups, demonstrating complete non−overlap of ranges and markedly different medians. Together, these visualizations support the use of the 15% cutoff as a data−driven and biologically meaningful stratification. [file Image2.jpeg]

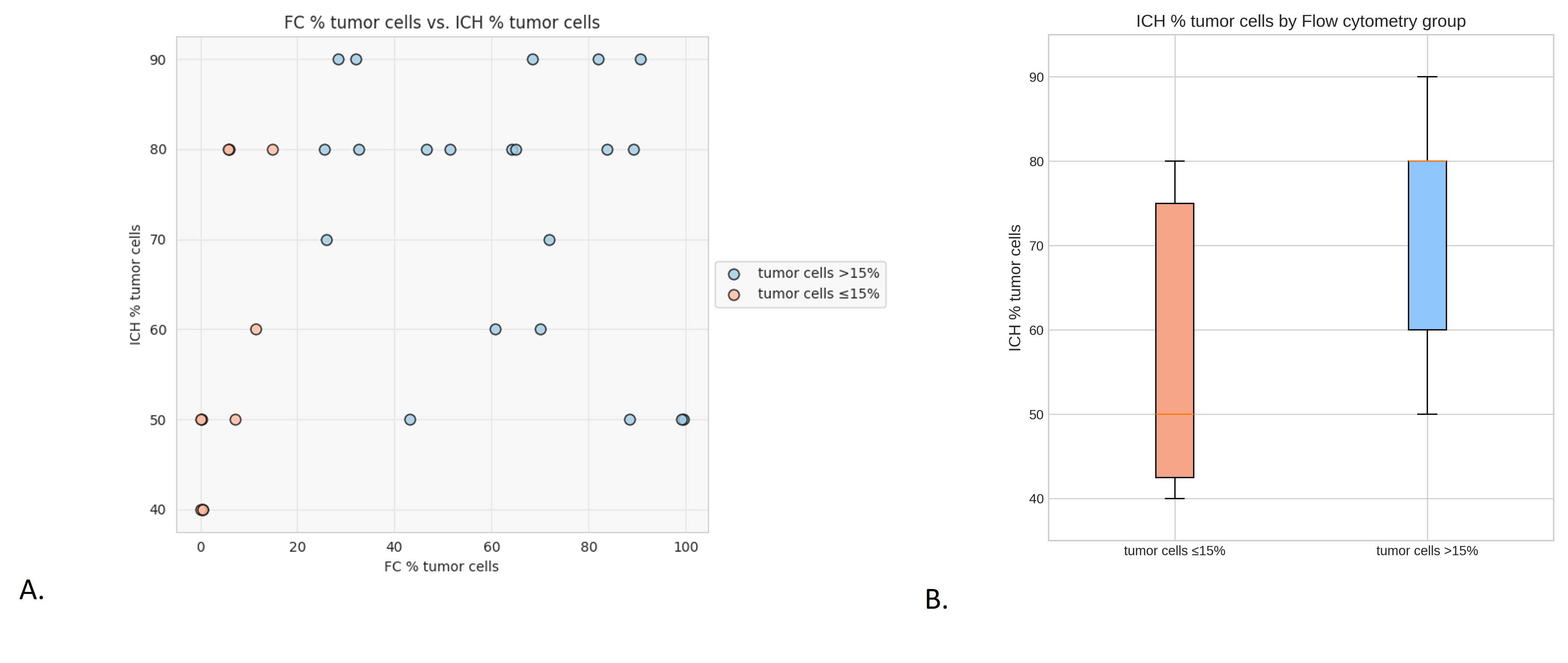

Supplement: Supplementary Figure 3 — (A) Scatterplot showing the relationship between flow cytometry–derived tumor cell percentage (FC %) and cytopathological tumor content (ICH %) in the subset of 31 lymph node (LN) samples with quantitative cell block assessment. A strong correlation was observed between the two modalities (Spearman ρ = 0.82, p < 0.00001), with three cases in which flow cytometry underestimated tumor content. Colors indicate samples with low (≤15%) and high (>15%) tumor cell content by flow cytometry. (B) Boxplot comparing ICH % tumor cell content between samples classified as ≤15% and >15% tumor cells by flow cytometry, using the same color scheme as in panel A. Samples with >15% tumor cells by flow cytometry demonstrated significantly higher cytopathological tumor fractions (median 80% vs 50%, Mann–Whitney p = 0.0132). [file Image3.jpeg]
