# Supplementary material for: Non-classical immune checkpoint CD137/CD137L and CD200/CD200R expressions are regulated by the tumor immune microenvironment in lymph node aspirates from lung cancer patients
Source: Front Immunol. 2026 May 26;17:1766726. doi: 10.3389/fimmu.2026.1766726 (PMC13246615; doi:10.3389/fimmu.2026.1766726)
Supplement: Supplementary file 4 [file Table1.docx]

Supplementary Table 1. Antibodies used for flow cytometry analysis of lymphocytes, tumor cells and expression of checkpoint molecules

| **Marker** | **Fluorochrome** | **Clone** | **Vendor** | **Purpose** | **Staining volumes** |
| --- | --- | --- | --- | --- | --- |
| CD45 | Krome Orange | J33 | Beckman Coulter | Leukocyte gating | 5 µl |
| EpCAM | A700 | EBA-1 | BioLegend | Tumor gating | 2 µl |
| Cytokeratin | BV421 | CAM5.2 | BD Biosciences | Tumor gating | 5 µl |
| CD3 | PC5.5 | UCHT1 | Beckman Coulter | Lymphocytes T gating | 5 µl |
| CD4 | BV650 | RPA-T4 | BioLegend | Lymphocytes T gating | 2 µl |
| CD8 | PE-DyLight™ 594 | MEM-31 | Sysmex | Lymphocytes T gating | 5 µl |
| CD137 | BV605 | 4B4-1 | BioLegend | Co-stimulation receptor | 2 µl |
| CD137L | PE-Cy7 | 5F4 | BioLegend | Ligand | 2 µl |
| CD200 | APC | OX104 | BioLegend | Co-stimulation receptor | 2 µl |
| CD200R | PE | OX-108 | BioLegend | Ligand | 2 µl |
| PD-1 | BB515 | EH12.1 | BD Biosciences | Checkpoint | 2 µl |
| PD-L1 | Alexa Fluor 750 | 8591R | Novus Biologicals™ | Checkpoint | 2 µl |
| PD-L2 | BV785 | 29E.2A3 | BioLegend | Checkpoint | 2 µl |
