# Supplementary material for: Non-classical immune checkpoint CD137/CD137L and CD200/CD200R expressions are regulated by the tumor immune microenvironment in lymph node aspirates from lung cancer patients
Source: Front Immunol. 2026 May 26;17:1766726. doi: 10.3389/fimmu.2026.1766726 (PMC13246615; doi:10.3389/fimmu.2026.1766726)
Supplement: Supplementary file 5 [file Table2.docx]

Supplementary Table 2. Sensitivity analysis of tumor‑cell percentage thresholds.

Sensitivity analysis of tumor‑cell percentage thresholds. Alternative cutoffs (10% and 20%) were applied to evaluate the robustness of the primary 15% threshold. The 20% cutoff produced identical group assignments, as no samples fell between 15% and 25%. The 10% cutoff reassigned only three samples, and all immunophenotypic trends remained qualitatively unchanged, indicating that the findings are not dependent on the specific choice of threshold.

| Threshold | Group definition | n (low) | n (high) | Notes |
| --- | --- | --- | --- | --- |
| **10%** | ≤10% vs >10% | 13 | 58 | 3 samples shift from low to high compared with 15% cutoff; immunophenotypic trends unchanged |
| **15%**  *(primary cutoff)* | ≤15% vs >15% | 16 | 55 | Natural bimodal separation; no values between 15–25% |
| **20%** | ≤20% vs >20% | 16 | 55 | Identical grouping to 15% cutoff because lowest value in high group is 25.5% |
