# Supplementary material for: Non-classical immune checkpoint CD137/CD137L and CD200/CD200R expressions are regulated by the tumor immune microenvironment in lymph node aspirates from lung cancer patients
Source: Front Immunol. 2026 May 26;17:1766726. doi: 10.3389/fimmu.2026.1766726 (PMC13246615; doi:10.3389/fimmu.2026.1766726)
Supplement: Supplementary file 6 [file Table3.docx]

Supplementary Table 3. Differences in the proportion of CD137/CD137L, CD200/200L and PD1/PD-L1, L2 expression on cell subpopulation in lymph nodes aspirates (LNs) between patients with non-small cell lung cancer (NSCLC) and small cell lung cancer (SCLC). Data expressed as median (Q1–Q3). * Indicates p statistically significant.

| Antigens: | % and GMF of antigens expression on population: | NSCLC median (Q1-Q3)  N= 38 | SCLC median (Q1-Q3)  N=33 | * p< 0.05 Mann-Whitney U test |
| --- | --- | --- | --- | --- |
| CD137L | Tumor cells | 55.6 (43.3-70.4) 7777 (4764-14472) | 51.0 (40.4-69.4) 8587 (4105-12165) | p= 0.4826 p= 0.9720 |
| CD137L | Lymphocytes | 27.8 (12.8-44.6) 7091 (2564-10892) | 30.5 (8.1-42.8) 6851 (2608-9023) | p= 0.6519 p= 0.6344 |
| CD137L | Lymphocyets T cells | 38.8 (8.3-57.4) 8702 (2536-15755) | 26.2 (9.6-75.1) 4189 (2051-17545) | p= 0.8952 p= 0.8012 |
| CD137L | CD4+ T cells | 74.8 (11.9-90.9) 7784 (2061-17391) | 40.3 (13.6-91.2) 7621 (2374-20334) | p= 0.6926 p= 0.5397 |
| CD137L | CD8+ T cells | 39.4 (14.8-69.0) 6824 (2838-11310) | 34.0 (10.2-62.6) 6580 (1618-13195) | p= 05144 p= 0.8812 |
| CD137 | Tumor cells | 28.6 (11.1-72.6) 1154 (731-2613) | 4.3 (2.0-26.8) 433 (278-659) | *p= 0.0007 *p<0.0001 |
| CD137 | Lymphocytes | 15.5 (3.2-25.6) 1502 (235-3926) | 18.2 (1.1-28.8) 996 (172-2126) | p= 0.9771 p=0.5473 |
| CD137 | Lymphocytes T cells | 17.8 (3.1-36.9) 2999 (426-5432) | 14.7 (1.3-40.0) 2239 (717-4413) | p= 0.7096 p= 0.7354 |
| CD137 | CD4+ T cells | 2.0 (0.5-6.5) 1531 (154-2662) | 1.7 (0.4-9.3) 1664 (151-2845) | p= 0.9772 p= 0.8591 |
| CD137 | CD8+ T cells | 51.8 (0.2-86.5) 7568 (850-16418) | 37.9 (0.0-84.8) 7658 (333-17551) | p= 0.6281 p=0.7879 |
| CD200R | Tumor cells | 6.1 (1.6-14.5) 715 (234-1334) | 0.8 (0.2-3.6) 611 (389-1172) | *p= 0.0003 p= 0.8792 |
| CD200R | Lymphocytes | 14.5 (4.6-28.3) 2321 (1525-5006) | 8.5 (3.2-27.3) 2379 (1255-4490) | p= 0.4525 p= 0.8054 |
| CD200R | Lymphocyets T cells | 15.9 (3.2-39.0) 2875 (1034-6013) | 5.8 (2.9-21.0) 2102 (928-5068) | p= 0.1187 p= 0.5171 |
| CD200R | CD4+ T cells | 5.4 (1.6-13.8) 2309 (993-4060) | 4.6 (1.7-9.3) 2602 (1423-3944) | p= 0.4595 p= 0.6674 |
| CD200R | CD8+ T cells | 67.7 (4.6-95.2) 8172 (1591-31436) | 10.2 (1.7-95.7) 3643 (1413-33455) | p= 0.5067 p=0.8436 |
| CD200 | Tumor cells | 3.9 (1.0-45.0) 433 (280-1311) | 29.1 (11.9-51.9) 1757 (965-3282) | *p= 0.0495 *p= 0.0034 |
| CD200 | Lymphocytes | 1.9 (0.5-5.3) 319 (190-759) | 1.8 (0.7-3.9) 295 (212-711) | p= 0.9133 p= 0.8681 |
| CD200 | Lymphocytes T cells | 1.6 (0.6-4.0) 319 (190-759) | 2.1 (0.4-7.2) 269 (129-522) | p= 0.5783 p= 0.6758 |
| CD200 | CD4+ T cells | 4.7 (1.4-12.7) 287 (159-601) | 2.9 (0.3-9.0) 346 (166-612) | p= 0.1006 p= 0.7702 |
| CD200 | CD8+ T cells | 7.3 (0.6-29.5) 272 (117-1464) | 5.2 (1.0-15.2) 228 (80-778) | p= 0.5699 p= 0.5946 |
| PD-L1 | Tumor cells | 18.7 (7.2-35.7) 797 (448-1037) | 4.5 (0.3-10.9) 435 (294-680) | *p= 0.0003 *p= 0.0045 |
| PD-L2 | Tumor cells | 45.1 (23.1-66.2) 948 (634-1488) | 14.9 (2.8-31.1) 525 (403-652) | *p= 0.0001 *p< 0.0001 |
| PD-1 | Lymphocytes T | 29.9 (20.6-45.8) 3198 (2278-4073) | 31.9 (22.4-41.7) 3295 (2182-3662) | p= 0.8951 p=0.5193 |
