# Supplementary material for: Non-classical immune checkpoint CD137/CD137L and CD200/CD200R expressions are regulated by the tumor immune microenvironment in lymph node aspirates from lung cancer patients
Source: Front Immunol. 2026 May 26;17:1766726. doi: 10.3389/fimmu.2026.1766726 (PMC13246615; doi:10.3389/fimmu.2026.1766726)
Supplement: Supplementary file 7 [file Table4.docx]

Supplementary Table 4. Differences in the proportion of CD137/CD137L, CD200/200L and PD1/PD-L1, L2 expression on cell subpopulation in lymph nodes aspirates (LNs) between patients with low and with high tumor fraction in the flow cytometry sample. Data expressed as median (Q1–Q3). * Indicates p statistically significant.

| Antigens: | % and GMF of antigens espression on population: | patients with low tumor fraction ≤ 15.0% median (Q1-Q3)  N= 16 | patients with high tumor fraction > 15.0% median (Q1-Q3)  N= 55 | * p< 0.05 Mann-Whitney U test |
| --- | --- | --- | --- | --- |
| CD137L | Tumor cells | 48.4 941-7-63.0) 6708 (4043-10447) | 56.4 (43.3-69.9) 8587 (4982-14058) | p= 0.3559 p= 0.1667 |
| CD137L | Lymphocytes | 20.9 (3.1-43.6) 4852 (1656-11686) | 30.5 (12.6-45.1) 7067 (3032-9842) | p= 0.2152 p= 0.5259 |
| CD137L | Lymphocyets T cells | 23.4 (2.0-50.6) 5398 (1282-15199) | 36.2 (11.1-66.2) 7595 (2352-17403) | p= 0.1451 p= 0.2941 |
| CD137L | CD4+ T cells | 51.0 (3.4-91.4) 7861 (1076-1759) | 72.7 (14.7-91.2) 7621 (2591-19307) | p= 0.3423 p= 0.3020 |
| CD137L | CD8+ T cells | 33.3 (4.0-57.1) 3728 (1836-8900) | 42.1 (14.3-69.0) 7694 (2838-12447) | p= 0.2734 p= 0.3479 |
| CD137 | Tumor cells | 47.7 (11.1-78.5) 2028 (962-3240) | 12.7 (2.2-40.3) 575 (311-897) | *p= 0.0178 *p<0.0001 |
| CD137 | Lymphocytes | 16.3 (0.8-23.9) 1480 (157-3259) | 16.9 (1.7-28.9) 1215 (235-3402) | p= 0.4653 p= 0.7904 |
| CD137 | Lymphocytes T cells | 17.7 (8.6-33.2) 2556 (145-5240) | 17.5 (3.1-41.4) 2605 (653-5881) | p= 0.6187 p= 0.8541 |
| CD137 | CD4+ T cells | 0.7 (0.4-1.7) 1567 (127-2623) | 2.4 (0.4-9.3) 1526 (151-5970) | p= 0.1557 p= 0.7695 |
| CD137 | CD8+ T cells | 39.6 (0.1-89.2) 7509 (706-17452) | 38.7 (0.1-82.9) 8051 (576-16418) | p= 0.5973 p= 0.6986 |
| CD200R | Tumor cells | 11.2 (1.8-14.5) 523 (196-1152) | 1.4 (0.4-10.3) 715 (378-1426) | *p= 0.0459 p= 0.3340 |
| CD200R | Lymphocytes | 10.9 (3.2-25.2) 1879 (576-4884) | 14.4 (4.5-28.9) 2379 (1322-4900) | p=0.4325 p= 0.4487 |
| CD200R | Lymphocyets T cells | 13.2 (3.0-32.4) 2172 (584-5615) | 9.1 (3.1-39.0) 2620 (1029-5297) | p= 0.8541 p= 0.9511 |
| CD200R | CD4+ T cells | 4.2 (1.2-9.9) 2310 (761-2851) | 5.4 (1.7-13.8) 2531 (1314-4070) | p= 0.3285 p= 0.1815 |
| CD200R | CD8+ T cells | 66.5 (2.6-97.6) 1471 (663-34004) | 25.7 (3.2-95.2) 5874 (1726-31436) | p= 07729 p= 0.9495 |
| CD200 | Tumor cells | 29.0 (1.2-75.5) 769 (313-2718) | 21.3 (1.4-50.0) 1095 (295-2829) | p= 0.4520 p= 0.7988 |
| CD200 | Lymphocytes | 2.0 (0.5-9.0) 397 (250-984) | 1.8 (0.7-4.2) 295 (175-711) | p= 0.9620 p= 0.2152 |
| CD200 | Lymphocytes T cells | 1.4 (0.7-8.9) 349 (98-976) | 1.7 (0.4-4.8) 233 (68-634) | p= 0.6091 p= 0.5807 |
| CD200 | CD4+ T cells | 9.1 (1.6-12.8) 331 (174-800) | 3.2 (0.4-11.1) 299 (129-612) | p= 0.1364 p= 0.5259 |
| CD200 | CD8+ T cells | 14.2 (1.2-52.8) 463 (231-2636) | 4.8 (0.6-16.8) 204 (82-802) | p= 0.0729 p= 0.1009 |
| PD-L1 | Tumor cells | 30.0 (9.4-42.3) 778 (393-1034) | 7.7 (1.1-23.2) 521 (310-967) | *p= 0.0193 p= 0.5901 |
| PD-L2 | Tumor cells | 52.3 (28.0-69.5) 1242 (676-1916) | 29.4 (7.6-46.2) 617 (444-904) | *p= 0.0275 *p= 0.0011 |
| PD-1 | Lymphocytes T | 23.0 (16.5-36.6) 2595 (2212-3469) | 33.4 923.7-43.5) 3373 (2611-4017) | p= 0.0918 p= 0.1971 |
